# Are local plants the best for ecosystem restoration? It depends on how you analyze the data

**DOI:** 10.1002/ece3.3585

**Published:** 2017-11-06

**Authors:** Anna Bucharova, Walter Durka, Norbert Hölzel, Johannes Kollmann, Stefan Michalski, Oliver Bossdorf

**Affiliations:** ^1^ Plant Evolutionary Ecology Institute of Evolution & Ecology University of Tübingen Tübingen Germany; ^2^ Nature Conservation and Landscape Ecology University of Freiburg Freiburg im Breisgau Germany; ^3^ Department of Community Ecology Helmholtz Centre for Environmental Research‐UFZ Halle Germany; ^4^ German Centre for Integrative Biodiversity Research (iDiv) Halle‐Jena‐Leipzig Leipzig Germany; ^5^ Biodiversity and Ecosystem Research Group Institute of Landscape Ecology University of Münster Münster Germany; ^6^ Department of Ecology & Ecosystem Management Restoration Ecology Technical University of Munich München Germany; ^7^ Norwegian Institute of Bioeconomy Research (NIBIO) Ås Norway

**Keywords:** experimental design, local adaptation, maladaptation, provenance, reciprocal transplant experiment, restoration ecology

## Abstract

One of the key questions in ecosystem restoration is the choice of the seed material for restoring plant communities. The most common strategy is to use local seed sources, based on the argument that many plants are locally adapted and thus local seed sources should provide the best restoration success. However, the evidence for local adaptation is inconsistent, and some of these inconsistencies may be due to different experimental approaches that have been used to test for local adaptation. We illustrate how conclusions about local adaptation depend on the experimental design and in particular on the method of data analysis. We used data from a multispecies reciprocal transplant experiment and analyzed them in three different ways: (1) comparing local vs. foreign plants within species and sites, corresponding to tests of the “local is best” paradigm in ecological restoration, (2) comparing sympatric vs. allopatric populations across sites but within species, and (3) comparing sympatric and allopatric populations across multiple species. These approaches reflect different experimental designs: While a local vs. foreign comparison can be done even in small experiments with a single species and site, the other two approaches require a reciprocal transplant experiment with one or multiple species, respectively. The three different analyses led to contrasting results. While the local/foreign approach indicated lack of local adaptation or even maladaptation, the more general sympatric/allopatric approach rather suggested local adaptation, and the most general cross‐species sympatric/allopatric test provided significant evidence for local adaptation. The analyses demonstrate how the design of experiments and methods of data analysis impact conclusions on the presence or absence of local adaptation. While small‐scale, single‐species experiments may be useful for identifying the appropriate seed material for a specific restoration project, general patterns can only be detected in reciprocal transplant experiments with multiple species and sites.

## 1 INTRODUCTION

Ecosystem restoration is now globally recognized as a key component in conservation programs and essential for the long‐term sustainability of our human‐dominated planet (Aronson & Alexander, 2013). In many cases, the critical first step of restoration projects is the re‐establishment of native plant communities, with active planting or sowing as common restoration tools.

While there is general agreement on the need for restoration, there is an intensive debate on the origin of the plant material for restoration (Breed, Stead, Ottewell, Gardner, & Lowe, 2013; Broadhurst et al., 2008; Bucharova, 2017; Sgrò, Lowe, & Hoffmann, 2011). One main strategy for the choice of seed material for ecosystem restoration is so‐called local provenancing (Hamilton, 2001) that is the use of local seed sources. It is based on a large body of evidence that populations of many plant species are adapted to their local environments, and therefore, local plants have a higher fitness than foreign ones (e.g., Becker, Colling, Dostal, Jakobsson, & Matthies, 2006; Bucharova et al., 2017; Joshi et al., 2001; Raabová, Münzbergová, & Fischer, 2011). Local provenancing is supported by many experts (Bucharova et al., 2017; Kiehl, Kirmer, Donath, Rasran, & Hölzel, 2010; McKay, Christian, Harrison, & Rice, 2005; Vander Mijnsbrugge, Bischoff, & Smith, 2010), but it has also been questioned (e.g., Breed et al., 2013; Broadhurst et al., 2008; Crowe & Parker, 2008; Jones, 2013; Sgrò et al., 2011), because local adaptation is not ubiquitous and local plants do not always perform better than all other ones. Meta‐analyses of local adaptation studies found strict evidence of local adaptation in about half of them (Hereford, 2009; Leimu & Fischer, 2008).

The most rigorous method of testing for local adaptation is reciprocal transplant experiments (Blanquart, Kaltz, Nuismer, & Gandon, 2013). Most knowledge on local adaptation comes from such studies of individual species (e.g., Bischoff & Trémulot, 2011; Mendola, Baer, Johnson, & Maricle, 2015; Mathiasen & Premoli, 2016; Evans et al., 2016; Hirst, Sexton, & Hoffmann, 2016; Lu, Parker, Colombo, Man, & Baeten, 2016 and many others). While a classical reciprocal transplant experiment involves at least two origins of a single species transplanted reciprocally to both sites of origin, some studies simplified this design to only multiple origins in one site (Gellie, Breed, Thurgate, Kennedy, & Lowe, 2016; Hancock, Leishman, & Hughes, 2013), whereas others have extended it to multiple origins of multiple species in multiple sites (e.g., Bischoff et al., 2006; Bucharova et al., 2017; Carter & Blair, 2012; Joshi et al., 2001; Körner et al., 2016; Kramer, Larkin, & Fant, 2015).

Data from transplant experiments can be analyzed for local adaptation in three different ways, all of which are commonly used. In the following, we generally use the terminology of Blanquart et al. (2013). First, the “home vs. away” approach compares performance of plants in their local environment to their performance in other environments (Kawecki & Ebert, 2004). This approach has the lowest ability to detect local adaptation (Blanquart et al., 2013), and because it is the least relevant in the context of seed‐sourcing strategies for ecosystem restoration, we do not consider it further. Second, the “local vs. foreign” method compares performance of local plants with that of foreign plants in the local environment (Blanquart et al., 2013). This method in its strictest form was lately used to test the “local is best” paradigm for seed‐sourcing strategies in ecological restoration (Gellie et al., 2016; Hancock & Hughes, 2014). Third, the “sympatric vs. allopatric” approach (unfortunately sometimes called “home vs. away” in older studies) uses multiple local populations and compares the general performance of plants growing in their local environments (sympatric) with plants growing outside their local environments (allopatric). Only the last approach allows to detect general patterns in local adaptation as it is not site‐specific (Blanquart et al., 2013), as exemplified by Joshi et al. (2001), Bischoff et al. (2006), Hirst et al. (2016), or Bucharova et al. (2017). The analysis approach depends on the experimental design. Single‐site studies allow only local/foreign analyses, whereas reciprocal transplants enable both local/foreign and sympatric/allopatric analyses.

In this study, we focused on the potential effects of experimental design and method of data analysis on our conclusions about local adaptation. We used data from a recent reciprocal transplant experiment with multiple grassland species (Bucharova et al., 2017) and analyzed the data in three different ways: (1) comparing local vs. foreign plants within species and sites, (2) comparing sympatric vs. allopatric populations across sites but within species, and (3) comparing sympatric and allopatric populations across multiple species.

## 2 METHODS

### 2.1 Data

We used data from a multispecies transplant experiment that tested for regional adaptation in seven common Central European grassland species (Bucharova et al., 2017). Regional adaptation is a geographically broader analog of local adaptation (Knapp & Rice, 1994). For simplicity, we will refer to the better‐established term “local adaptation” throughout this article. The original study included eight origins per species, hereafter called ecotypes, transplanted in twelve replicates to four experimental gardens in Germany (Fig. S1). In May 2013, the seeds of each ecotype were germinated in a glasshouse and 12 seedlings per species and ecotype transplanted into pots filled with a standard potting soil (same pots and soil used in all sites) and placed outside in a randomized design. To avoid drought‐related mortality, the pots were watered when needed during the hottest summer period. The plants were harvested in September 2013; for details, see Bucharova et al. (2017).

Here, we simplified the dataset to a full reciprocal design and used only the four ecotypes of the four regions where the experimental gardens were located. Further, we used only six of the seven species, because one species, *Knautia arvensis*, turned out to have different ploidy levels across regions (Durka et al., 2017), which should rather be treated as different taxa (Kolár et al., 2009). The final dataset contained six perennial species: *Arrhenatherum elatius* (L.) P.B. ex J. et C. Presl, *Centaurea jacea* L., *Daucus carota* L.*, Galium album* Mill., *Hypochaeris radicata* L., and *Lychnis flos‐cuculi* (L.) Greuter & Burdet (details in Bucharova et al., 2017). For two species (*Galium* and *Lychnis*), only seeds from three regions were available, resulting in an overall number of 22 species x garden combinations and 984 plants. As a measure of overall plant performance and proxy for fitness, we used the dry aboveground biomass of plants. Admittedly, biomass may not be the most precise estimate of true fitness, but the main focus of this study is on the method of analysis, which can be applied to any measure of fitness.

### 2.2 Data analysis

First, we analyzed the data using the local/foreign approach, that is, individually for each garden × species combination. For each garden and species, we fitted a linear model with ecotype as explanatory variable. We compared the differences between ecotypes using the Tukey's test in the R package *multcomp*. Cases where the local ecotypes outperformed all nonlocal ecotypes (“local is best”) were considered evidence for local adaptation, while cases where at least one of the nonlocal ecotypes significantly outperformed the local one were considered evidence of maladaptation (Hancock et al., 2013).

Second, we analyzed the data using the sympatric/allopatric approach separately for each species using linear mixed models. For each species, the model included garden identity and sympatry/allopatry as fixed factors and ecotype identity as a random factor. To obtain a quantitative measure of the relative advantage of local plants over nonlocal ones, we used the effect sizes, expressed as the percentage difference between plants growing in sympatry vs. allopatry. The original sympatry/allopatry approach recommends including ecotype identity as a fixed factor to correct for ecotype quality (Blanquart et al., 2013). This makes sense when investigating evolutionary processes, but here we focused on the identification of the most suitable material for restoration, and differences in ecotype quality are a reality that must be considered in such practical decisions. Therefore, we included ecotype identity as a random factor, to correct for the variance but not for the mean of ecotype performance (Korner‐Nievergelt et al., 2015).

Third, we carried out a sympatric/allopatric analysis across all species to test for a general pattern of local adaptation. Because the species differed in size, we standardized all biomasses by subtracting the mean and dividing by standard deviation within each species prior to the analysis. The biomass of each species thus had a mean of 0 and a standard deviation of 1. We used linear mixed models with garden identity and sympatry/allopatry as fixed factors and ecotype identity nested within species as random factors.

To evaluate model fit, we calculated *R* ^2^ for all models. For the simple linear models (first approach), we obtained *R* ^2^ directly from the model summaries. For the linear mixed models (second and third approach), we calculated approximate conditional *R* ^2^, that is, a proportion of variance explained by both random and fixed factors, using the *MuMIn* package in R.

To obtain a measure of confidence of the effects sizes derived from the mixed models (second and third approach), we used Bayesian model inference (Korner‐Nievergelt et al., 2015). From the maximum‐likelihood estimates of each model and using a noninformative prior, we calculated the posterior distributions represented by 10,000 random samples from this distribution (*sim* function in the R package *arm*; for details see Korner‐Nievergelt et al. (2015)). From these posterior distributions, we then derived 95% credible intervals, the Bayesian analogues of confidence intervals. Finally, we calculated the posterior probability that plants growing in sympatry produce more biomass than plants growing in allopatry. If a posterior probability was greater than 0.95, we considered this evidence of local adaptation, and if the probability was below 0.05, we considered the results as evidence for maladaptation. This approach is generally more reliable for testing the significance of fixed factors in GLMM than the *P*‐values of classical likelihood‐ratio tests (Bolker et al., 2009).

Admittedly, a model that tests sympatric/allopatric across all the species has greater statistical power than models testing the same in individual species, that is, using only subsets of the same data. To be sure that our results were not driven solely by differences in statistical power, we resampled the data in the sympatric/allopatric analysis so that the number of replicates equaled that of the individual species models, and we used these data to refit the cross‐species model. We repeated this procedure 10,000 times and calculated the proportion of permutations in which the effect size based on the resampled data pointed toward an advantage of plants grown in sympatry.

## 3 RESULTS

**Figure 1:**
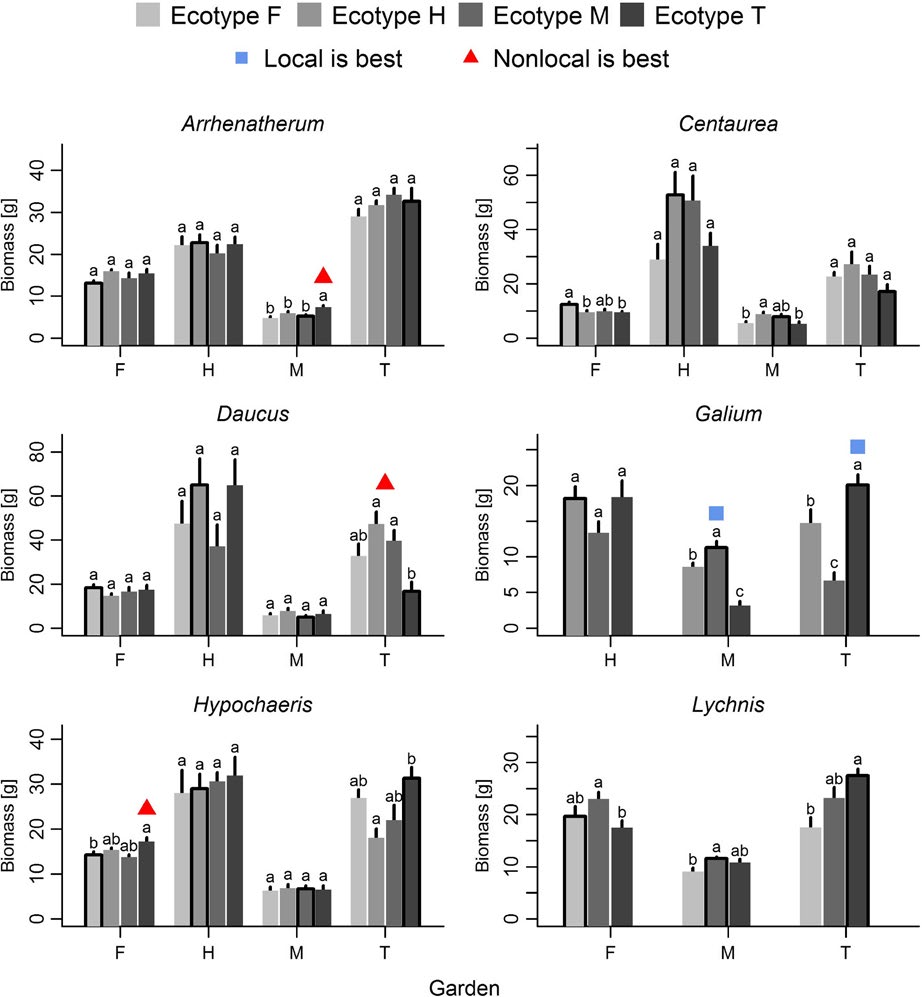
The performances of different ecotypes in different gardens, separately for each species (mean ± *SE*). Capital letters indicate the four study sites, each in a different region: F = Freising, H = Halle, M = Münster, T = Tübingen. Bars with thick borders indicate the respective local ecotype in a site. Different small letters above the bars indicate significant differences between ecotypes (*p *<* *.05). Blue quadrats represent cases where local ecotypes significantly outperformed all nonlocal ecotypes in a site, supporting “local is best.” Red triangles are cases where local ecotypes were significantly outperformed by at least one nonlocal ecotype, supporting “nonlocal is best.” For statistical details, see Tables S1 and S2

**Figure 2:**
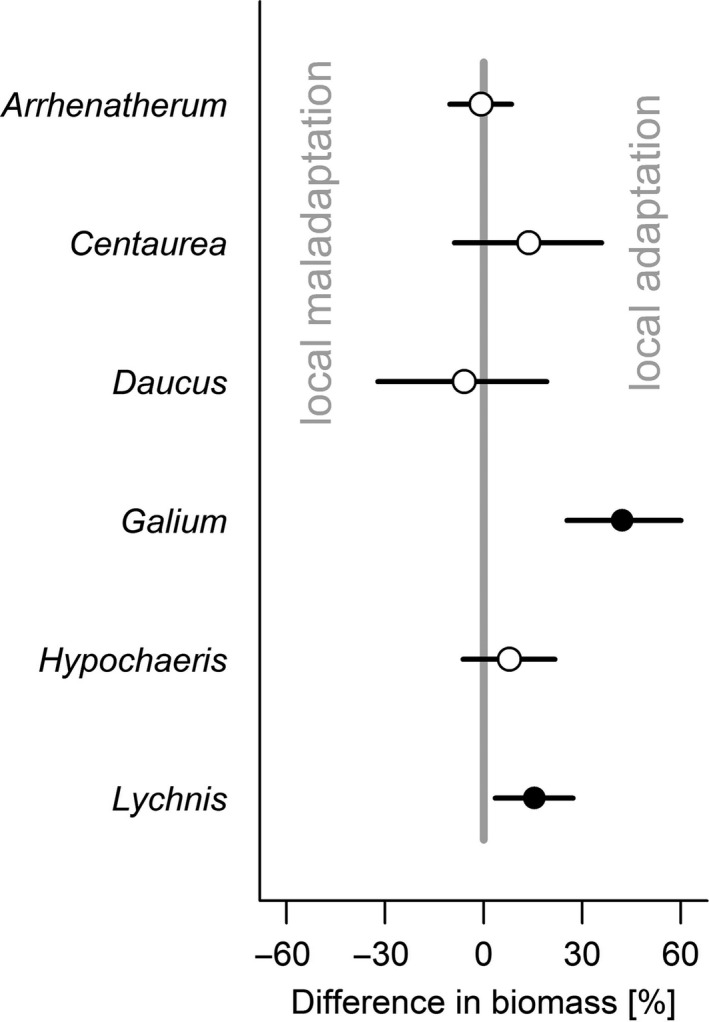
The difference in percentage biomass between plants growing in sympatry vs. allopatry, with positive values indicating higher performance of plants growing in sympatry and negative values indicating higher performance in allopatry. Dots are effect size, and lines are credible intervals. Significant results are indicated by filled dots. The posterior probabilities that the values are positive: *Arrhenatherum p *=* *.427, *Centaurea p *=* *.883, *Daucus p *=* *.332, *Galium p *>* *.999, *Hypochaeris p *=* *.862*,* and *Lychnis p = .996*. See Table S2 for the model fits of each model

**Figure 3:**
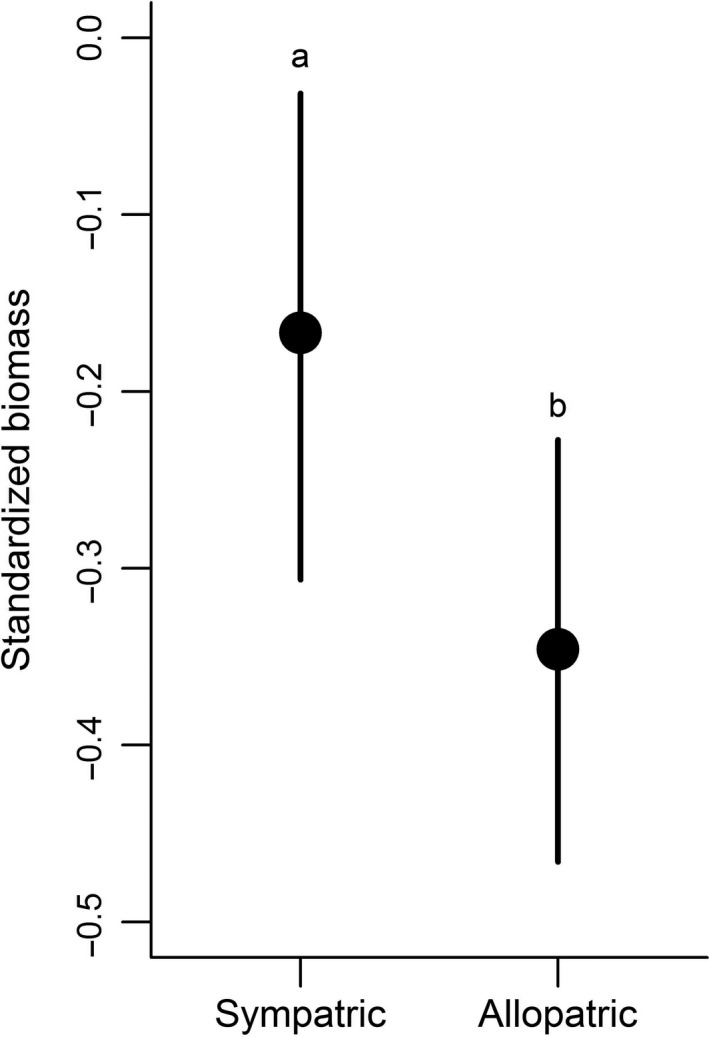
The performance of sympatric (local) vs. allopatric (nonlocal) ecotypes across all species and gardens. The plotted values represent the fitted values of the models; vertical bars represent credible intervals, and different letters indicate significant differences. The posterior probability that plants in sympatry produce more biomass than plants in allopatry is *p *=* *.999. Model fit in Table S2

Using the simple local/foreign approach, we found evidence for “local is best,” that is, the local ecotype performs significantly better than all foreign ecotypes, only for one species and this only in two gardens. On the other hand, we found support for “nonlocal is best,” that is, at least one nonlocal ecotype is significantly better than the local one, for each of three species in one garden. For the majority of species and garden combinations, there were no significant differences between local and nonlocal ecotypes (Figure 1). When we analyzed the same data using the sympatric/allopatric approach separately for each species, we found that in two of six species the plants growing in sympatry (i.e., local) produced significantly more biomass than plants growing in allopatry (i.e., nonlocal), and in the other four species, there were no significant differences (Figure 2). When we analyzed all data together, we found that across all six species plants growing in sympatry (i.e., local ecotypes) produced significantly more biomass than plants growing in allopatry (Figure 3). Resampling the dataset provided similar results for the sympatry/allopatry test: In 95% of the permutations, the effect size pointed toward an advantage of plants growing in sympatry.

## 4 DISCUSSION

The debate on seed‐sourcing strategies for ecosystem restoration largely depends on the outcome of studies testing for local adaptation (Breed et al., 2013; Broadhurst et al., 2008). Here, we demonstrated that our ability to detect local adaptation critically depends on the experimental design and method of data analysis. While in our dataset, the simplest, but frequently used, “local is best” approach rather indicated disadvantages of local ecotypes, the most comprehensive test provided evidence for local adaptation.

### 4.1 Why are the results different?

When we analyzed the data as it would be done in a single‐site experiment (Gellie et al., 2016; Hancock et al., 2013), or in a multiple‐site transplant experiment analyzed with the local/foreign approach (the strict definition of local adaptation in the meta‐analysis of Leimu and Fischer (2008)), the results suggested a more frequent disadvantage than advantage of local ecotypes (Figure 1). It is important to understand that this approach is very conservative, because to accept “local is best,” each local ecotype must perform better than all nonlocal ecotypes, while to reject it, it is sufficient that only one of the nonlocal ecotypes performs better than the local one. Under such strict criteria, the “local is best” paradigm is more likely to be rejected than to be accepted.

While the local/foreign analysis rather pointed to a lack of local adaptation or even local maladaptation, the sympatry/allopatry approach more frequently indicated local adaptation, and never maladaptation. This discrepancy is likely because the first approach compares values for specific ecotypes in individual gardens, whereas the latter one compares *averages* across multiple gardens and ecotypes (Blanquart et al., 2013). Thus, the local ecotype does not always need to be the best one, and even if individual nonlocal ecotypes perform better in some places, the local ecotypes could still be *on average* a better choice than the *average* nonlocal ecotype.

The evidence for local adaptation was stronger when sympatry/allopatry was evaluated across all species, than when it was tested for each species separately (Figures 2 and 3). Part of this might be explained by the increase of statistical power in multispecies compared to single‐species analyses. However, given the 99.9% posterior probability of difference in the multispecies test, the results of the permutation test on resampled data, and the fact that in the single‐species analyses several nonsignificant ones also pointed toward local adaptation, we are confident that the significant multispecies comparison described a true general pattern across the tested species.

### 4.2 Testing for local adaptation in a restoration context

The choice of experimental design and the data analysis approach decisively influence the interpretation of studies that test for local adaptation in a restoration context. Simple experiments that transplant multiple ecotypes into only one experimental site (Gellie et al., 2016; Hancock et al., 2013) can be interpreted solely in their local context in testing whether the *particular ecotype* performs better at this *particular site*. Such studies may be useful for identification of the best plant material for a specific restoration project, but their results should not be generalized beyond the particular site and species.

Results that can be generalized at species level require reciprocal transplant experiments, multiple planting sites, and an appropriate data analysis. The local/foreign method at a single site is inappropriate here, because it may not reveal the general pattern of local adaptation. In contrast, the sympatric/allopatric approach combines the information from all transplant sites into one model (Blanquart et al., 2013). If local adaptation is confirmed, as in this study, local ecotypes will perform *on average* better than a randomly chosen nonlocal ecotype. However, this does not mean that local ecotypes will always perform best. Ideally, we would be able to distinguish between the nonlocal ecotypes and predict which of them will perform better than the local one. Studies using indices of geographical distance or environmental similarity (Bischoff et al., 2006; Bucharova et al., 2017; Fenster & Galloway, 2000) suggest to use the closest ecotype, or an ecotype from a site with most similar environmental conditions, that is, the local one. Consequently, the use of the local ecotype is the safest choice.

Similar to the prediction of within‐species patterns from single‐site studies, it is impossible to predict a general, multiple‐species pattern from single‐species studies, because a lack of local adaptation in individual species does not mean a general lack of local adaptation in the study system (compare Figures 2 and 3). The best approach to test for general patterns of local adaptation, for example, for communities of species‐rich grasslands, is to involve multiple species and multiple transplant sites, ideally followed over several years (van Kleunen, Dawson, Bossdorf, & Fischer, 2014). Only complex reciprocal transplant experiments will provide the knowledge necessary to develop general guidelines for seed‐sourcing strategies in ecological restoration.

## CONFLICT OF INTEREST

None declared.

## AUTHOR CONTRIBUTION

AB conceived the idea, performed the analyses, and wrote the first draft. WD, NH, JK, SM, and OB contributed to discussion and manuscript writing.
