# Supplementary material for: Are local plants the best for ecosystem restoration? It depends on how you analyze the data
Source: Ecol Evol. 2017 Nov 6;7(24):10683–9. doi: 10.1002/ece3.3585 (PMC5743477; doi:10.1002/ece3.3585)

**Table S1**: Pairwise differences between ecotype (eco) performances within individual gardens. Significant values (*P* < 0.05) are in bold, differences related to local ecotypes are in black frames. Local ecotypes have the same letter as the respective gardens.

|  | **Garden F** | | | |  | **Garden H** | | | |  | **Garden M** | | | |  | **Garden T** | | | |
| --- | --- | --- | --- | --- | --- | --- | --- | --- | --- | --- | --- | --- | --- | --- | --- | --- | --- | --- | --- |
| ***Arrhenatherum*** |  | **ecoF** | **ecoH** | **ecoM** |  |  | **ecoF** | **ecoH** | **ecoM** |  |  | **ecoF** | **ecoH** | **ecoM** |  |  | **ecoF** | **ecoH** | **ecoM** |
|  | **ecoH** | 0.153 | - | - |  | **ecoH** | 0.997 | - | - |  | **ecoH** | 0.199 | - | - |  | **ecoH** | 0.786 | - | - |
|  | **ecoM** | 0.806 | 0.582 | - |  | **ecoM** | 0.887 | 0.785 | - |  | **ecoM** | 0.871 | 0.597 | - |  | **ecoM** | 0.286 | 0.822 | - |
|  | **ecoT** | 0.276 | 0.982 | 0.791 |  | **ecoT** | >0.999 | 0.999 | 0.861 |  | **ecoT** | **<0.001** | **0.017** | **0.003** |  | **ecoT** | 0.592 | 0.988 | 0.949 |
|  |  |  |  |  |  |  |  |  |  |  |  |  |  |  |  |  |  |  |  |
| ***Centaurea*** |  | **ecoF** | **ecoH** | **ecoM** |  |  | **ecoF** | **ecoH** | **ecoM** |  |  | **ecoF** | **ecoH** | **ecoM** |  |  | **ecoF** | **ecoH** | **ecoM** |
|  | **ecoH** | **0.043** | - | - |  | **ecoH** | 0.130 | - | - |  | **ecoH** | **0.030** | - | - |  | **ecoH** | 0.729 | - | - |
|  | **ecoM** | 0.107 | 0.977 | - |  | **ecoM** | 0.191 | 0.997 | - |  | **ecoM** | 0.192 | 0.832 | - |  | **ecoM** | 0.998 | 0.847 | - |
|  | **ecoT** | **0.041** | > 0.999 | 0.974 |  | **ecoT** | 0.964 | 0.266 | 0.371 |  | **ecoT** | 0.997 | **0.018** | 0.128 |  | **ecoT** | 0.623 | 0.127 | 0.550 |
|  |  |  |  |  |  |  |  |  |  |  |  |  |  |  |  |  |  |  |  |
| ***Daucus*** |  | **ecoF** | **ecoH** | **ecoM** |  |  | **ecoF** | **ecoH** | **ecoM** |  |  | **ecoF** | **ecoH** | **ecoM** |  |  | **ecoF** | **ecoH** | **ecoM** |
|  | **ecoH** | 0.434 | - | - |  | **ecoH** | 0.652 | - | - |  | **ecoH** | 0.698 | - | - |  | **ecoH** | 0.189 | - | - |
|  | **ecoM** | 0.896 | 0.845 | - |  | **ecoM** | 0.907 | 0.270 | - |  | **ecoM** | 0.947 | 0.384 | - |  | **ecoM** | 0.773 | 0.701 | - |
|  | **ecoT** | 0.989 | 0.630 | 0.981 |  | **ecoT** | 0.674 | > 0.999 | 0.294 |  | **ecoT** | 0.989 | 0.868 | 0.827 |  | **ecoT** | 0.133 | **<0.001** | **0.015** |
|  |  |  |  |  |  |  |  |  |  |  |  |  |  |  |  |  |  |  |  |
| ***Galium*** |  | **ecoF** | **ecoH** | **ecoM** |  |  | **ecoF** | **ecoH** | **ecoM** |  |  | **ecoF** | **ecoH** | **ecoM** |  |  | **ecoF** | **ecoH** | **ecoM** |
|  | **ecoH** | **-** | - | - |  | **ecoH** | - | - | - |  | **ecoH** | - | - | - |  | **ecoH** | - | - | - |
|  | **ecoM** | **-** | - | - |  | **ecoM** | - | 0.195 | - |  | **ecoM** | - | **0.029** | - |  | **ecoM** | - | **0.003** | - |
|  | **ecoT** | **-** | - | - |  | **ecoT** | - | 0.998 | 0.175 |  | **ecoT** | - | **<0.001** | **<0.001** |  | **ecoT** | - | **0.048** | **<0.001** |

|  | **Garden F** | | | |  | **Garden H** | | | |  | **Garden M** | | | |  | **Garden T** | | | |
| --- | --- | --- | --- | --- | --- | --- | --- | --- | --- | --- | --- | --- | --- | --- | --- | --- | --- | --- | --- |
| ***Hypochaeris*** |  | **ecoF** | **ecoH** | **ecoM** |  |  | **ecoF** | **ecoH** | **ecoM** |  |  | **ecoF** | **ecoH** | **ecoM** |  |  | **ecoF** | **ecoH** | **ecoM** |
|  | **ecoH** | **0.081** | - | - |  | **ecoH** | 0.998 | - | - |  | **ecoH** | 0.955 | - | - |  | **ecoH** | 0.062 | - | - |
|  | **ecoM** | 0.929 | >0.999 | - |  | **ecoM** | 0.962 | 0.991 | - |  | **ecoM** | 0.981 | 0.999 | - |  | **ecoM** | 0.476 | 0.684 | - |
|  | **ecoT** | >0.999 | **0.004** | **0.076** |  | **ecoT** | 0.882 | 0.950 | 0.994 |  | **ecoT** | 0.997 | 0.989 | 0.998 |  | **ecoT** | 0.599 | **0.004** | 0.059 |
|  |  |  |  |  |  |  |  |  |  |  |  |  |  |  |  |  |  |  |  |
| ***Lychnis*** |  | **ecoF** | **ecoH** | **ecoM** |  |  | **ecoF** | **ecoH** | **ecoM** |  |  | **ecoF** | **ecoH** | **ecoM** |  |  | **ecoF** | **ecoH** | **ecoM** |
|  | **ecoH** | **-** | - | - |  | **ecoH** | - | - | - |  | **ecoH** | **-** | - | - |  | **ecoH** | **-** | - | - |
|  | **ecoM** | 0.283 | - | - |  | **ecoM** | - | - | - |  | **ecoM** | **0.012** | - | - |  | **ecoM** | 0.215 | - | - |
|  | **ecoT** | 0.577 | **-** | **0.040** |  | **ecoT** | - | - | - |  | **ecoT** | 0.141 | **-** | 0.651 |  | **ecoT** | **0.002** | **-** | 0.078 |

**Table S2:** Model fit, expressed as R^2^, for all models. In the mixed models (across sites, within and across species), the values are conditional R^2^, which indicates variability explained by both fixed and random factors.

|  | within species, within site | | | |  | across sites, within species |  | across sites, across species |
| --- | --- | --- | --- | --- | --- | --- | --- | --- |
| Species | garden F | garden H | garden M | Garden T |  |  |  |  |
| *Arrhenatherum* | 0.117 | 0.023 | 0.349 | 0.072 |  | 0.772 |  | 0.457 |
| *Centaurea* | 0.191 | 0.155 | 0.240 | 0.109 |  | 0.511 |  |  |
| *Daucus* | 0.055 | 0.101 | 0.060 | 0.309 |  | 0.411 |  |  |
| *Galium* | - | 0.118 | 0.667 | 0.537 |  | 0.423 |  |  |
| *Hypochaeris* | 0.230 | 0.014 | 0.007 | 0.283 |  | 0.566 |  |  |
| *Lychnis* | 0.167 | - | 0.246 | 0.323 |  | 0.547 |  |  |

**Figure S1**: Location of origins (circles) and experimental gardens (open quadrats) (Bucharova *et al.* 2017).


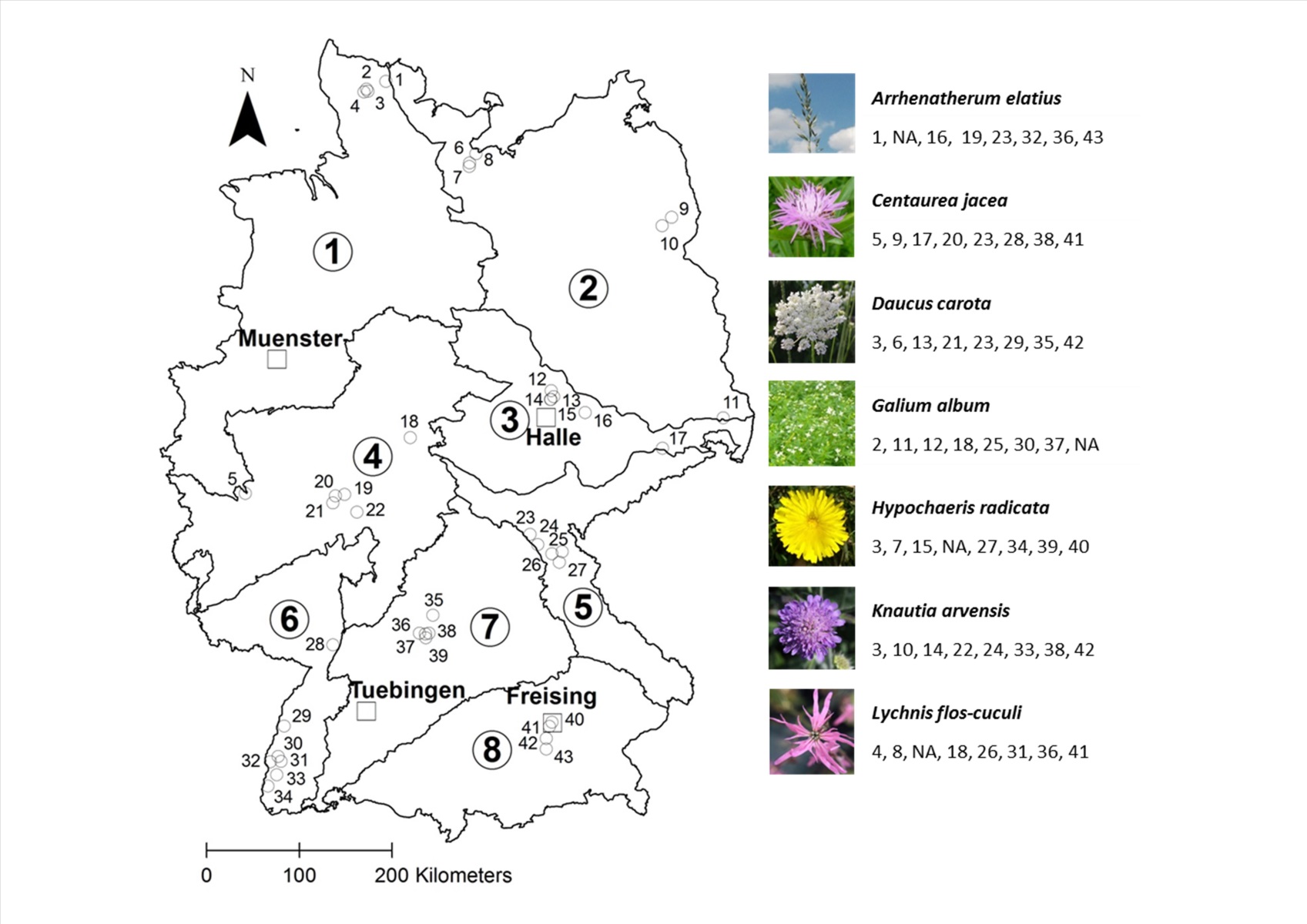

Supplement: Supplementary file 1 [file ECE3-7-10683-s001.docx]
